# Knowledge, attitude and practices of frontline health workers in relation to detection of brucellosis in rural settings of Tanzania: a cross-sectional study

**DOI:** 10.1186/s42522-021-00056-5

**Published:** 2022-01-04

**Authors:** Belinda Joseph Mligo, Calvin Sindato, Richard B. Yapi, Coletha Mathew, Ernatus M. Mkupasi, Rudovick R. Kazwala, Esron D. Karimuribo

**Affiliations:** 1 College of Veterinary Medicine and Biomedical Sciences, Sokoine University of Agriculture, P.O. Box 3015, Morogoro, Tanzania; 2 SACIDS Foundation for One Health, Sokoine University of Agriculture, P.O. Box 3297, Morogoro, Tanzania; 3 National Institute for Medical Research, Tabora Research Centre, Tabora, Tanzania; 4 Centre Suisse de Recherches Scientifiques en Côte d’Ivoire, Abidjan, Côte d’Ivoire; 5 Centre d’Entomologie Médicale et Vétérinaire, Université Alassane Ouattara, Bouaké, Côte d’Ivoire

**Keywords:** Brucellosis, Knowledge, Attitude, Practices, Frontline health workers, Tanzania

## Abstract

**Background:**

Brucellosis an important zoonotic disease worldwide, which frequently presents as an undifferentiated febrile illness with otherwise varied and non-specific clinical manifestations. Despite its importance, there are few reports on its awareness among frontline health workers. This study aimed at assessing the baseline knowledge, attitude and practice (KAP) related to detection and management of brucellosis among frontline health workers (FHWs) namely; healthcare workers (HWs) and community health workers (CHWs).

**Methods:**

A cross-sectional study was conducted from December 2019 to January 2020 in Kilosa and Chalinze districts of Tanzania. Data on demographic characteristics, knowledge, attitude and practices regarding brucellosis were collected from the study participants using a structured questionnaire. Interviews were conducted with 32 HWs and 32 CHWs who were systematically selected in study districts. Chi square/fisher Exact was used to assess the association between sociodemographic variables and those related to knowledge, attitude and practices.

**Results:**

Overall, a total of 30 (93.8%) HWs and nine (28.1%) CHWs from the study districts heard about brucellosis, with (34.4%) of HWs having knowledge about the causative organism. Overall, knowledge showed almost half (46.9%) HWs and (28.1%) CHWs were aware of the symptoms, clinical signs, diagnosis and control regarding brucellosis. Knowledge difference was statistically significant with HWs’ age (*p* = 0.016)*.* Almost half (46.9%) HWs and less than quarter (12.5%) CHWs had good practices regarding brucellosis control. Almost three quarters (71.9%) of HWs and (21.9%) CHWs had positive attitude regarding brucellosis control; overall attitude was statistically significant with CHWs age (*p* = 0.028) and education level (*p* = 0.024)*.* Lack of awareness and unavailability of diagnostic tools were the main challenges faced by FHWs in the two districts.

**Conclusion:**

The majority of participants were not aware of human brucellosis. Moreover, their overall knowledge was inadequate and the common practices were diagnostic tools, and adequate knowledge to manage brucellosis cases. These findings highlight the need to strengthen frontline health workers knowledge, practices and diagnostic capacities related to brucellosis.

**Supplementary Information:**

The online version contains supplementary material available at 10.1186/s42522-021-00056-5.

## Introduction

### Background

Brucellosis is one of the most common and highly infectious zoonotic disease globally [1]. Its occurrence results in food insecurity with devastating health and productivity effects, mainly in low- and middle-income countries [2, 3]. The disease has health and economic complications in both animals and humans [4]. It is caused by various species of bacteria of the genus *Brucella*. It can infect livestock including cattle, sheep, goats and pigs as well as other mammals and humans [5]. In most cases, the infected animals are the major sources of infection to humans [6]. Brucellosis is transmitted to humans mainly through consumption of products from infected animals including blood, meat and unpasteuralized milk, inhalation of contaminated airborne particulates, and/or direct contact with infected animals or their products [2]. It is also an occupational disease affecting veterinarians, farmers, abattoir workers and laboratory workers mainly through handling of infected animals and aborted foetuses or placentae. Clinical manifestations in humans include fever, joint pain, night sweating, loss of appetite, chills, weight loss and general fatigue. In women, brucellosis may also cause abortion [7, 8]. The disease is spread amongst animals when the infected animal aborts or gives birth. The bacteria causing the disease can survive outside the animal in the environment for several months, especially in cool moist conditions remaining infectious to other animals which become infected by ingesting the bacteria. The bacteria also infect the female animal’s udder spreading infections through milk [9]. As it is with humans, animals can acquire infections through cuts in the skin, or through mucous membranes. The disease affects wild animals that serve as reservoirs without obvious clinical manifestations, thus complicating the eradication efforts [9]. The major manifestation of brucellosis in animals is abortion, reduced fertility, weak offspring and reduction in milk production although, in some cases, infected animals may not show any clinical signs [10, 11].

In Tanzania, brucellosis was first reported in 1927 following an outbreak of abortion in cattle in Arusha region [12]. Since that time, many studies have been conducted, mostly in animals indicating variation in disease seroprevalence in different locations of the country [13, 14]. In a relatively few studies conducted in humans in Tanzania, the reported prevalence of the disease in northern Tanzania was 8.3% in 2005, 3.5% in 2012 and 6.1% in 2020 [12, 15, 16] and in eastern Tanzania, in Kilosa district a prevalence of 15.4% was reported in 2015 [17]. This indicates a wide geographical spread of the disease and small-scale prevalence studies are thus likely to be an underestimation of the real situation [18, 19].

Diagnosis of human brucellosis remains challenging mainly because of inadequate awareness of the disease among healthcare workers as well as the overlapping clinical manifestations with malaria that often results in its misdiagnosis [20]. In addition, less attention is paid by the medical practitioners to brucellosis as a cause of illness in the course of clinical assessment at the primary health care facilities, contributing to under/mis-diagnosis of the disease [21]. For effective control of the disease, adequate knowledge of causes, mode of transmission, signs and symptoms, as well as appropriate practices and positive attitude relating to the disease are required [22]. Several studies have shown limited awareness among healthcare providers on zoonotic diseases in Tanzania [18, 23, 24]. Brucellosis largely remains undetected and misdiagnosed as other causes of febrile illnesses [25]. For instance, a study carried out in northern Tanzania from 2012 to 2014, revealed that up 50 (8.9%) of 562 febrile patients enrolled in hospitals after receiving health care had brucellosis, that had not considered during their diagnosis and hospitalization [18]. It has been observed that despite the prevalence of brucellosis in Tanzania, clinicians still misdiagnose and manage it as malaria [21]. Involvement of both healthcare workers and community health workers provides an opportunity for collaboration for early detection and response to brucellosis in Tanzania [24, 26].

This paper aimed to assess frontline health workers’ ability to deal with human brucellosis. A cross-sectional study was conducted in the purposively selected districts of Kilosa and Chalinze. The findings of this study are anticipated to provide guidance on improved prevention, control and management of the disease.

## Materials and methods

### Study area

**Fig. 1:**
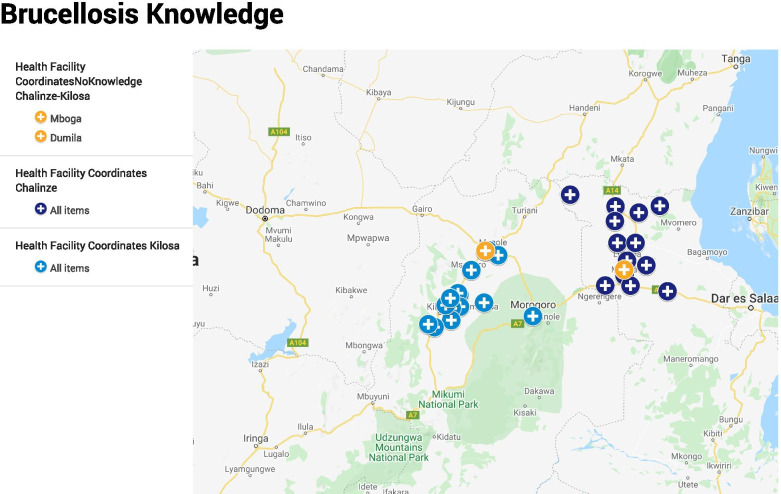
The map showing selected ward health facilities in Kilosa and Chalinze districts in Tanzania. Map created by ArcView GIS software version 3.2. Shapefiles for administrative boundaries from the 2012 census were sourced from the Tanzania National Bureau of Statistics

This study was carried out in Kilosa district in Morogoro region and Chalinze district in Pwani region, both located in the eastern part of Tanzania (Fig. 1). The districts were purposively selected as the areas with high population of pastoralist communities, typically keeping large numbers of domestic ruminants (cattle, sheep and goats). Previous studies in the region have reported high prevalence of brucellosis and/or low community knowledge and awareness of zoonotic diseases [27]. In both districts, agro-pastoralism is the main economic and income generating activity [28]. Administratively, Tanzania is divided into regions and each region is subdivided into districts. The districts are sub-divided into divisions and further into wards. The wards are further subdivided, for urban wards into streets and for rural wards into villages. Each ward is served with at least one primary health care facility (commonly a dispensary) and each village is served with at two Community Health Workers (CHWs) [29]. Figure 1 shows the health facility coverage included in the study.

### Study design and participants

From December 2019 to January 2020, a cross sectional study using an interview-based survey was conducted among frontline health workers. Based on available resources, our study involved a convenient overall sample of 64 stakeholders comprising an equal number of 32 health workers (HWs) from primary health care facilities and 32 CHWs from Kilosa and Chalinze districts. A purposive sampling approach was employed to select 16 wards in Kilosa and 16 in Chalinze based on perceived risk of brucellosis utilizing local knowledge and/or available records. In each ward, a primary health care facility serving the ward was selected from which the medical officer in-charge was invited to take part in the study. One CHW from the same village as the selected health facility was also invited to participate in the study.

### Data collection

A structured questionnaire, uploaded in the *AfyaData* app [30], was used to collect data related to knowledge, attitude and practices from the participants. The information collected included socio demographic characteristics (age, gender, education, length of stay in position and experience in workstation), knowledge of brucellosis (causes, mode of transmission, symptoms, diagnosis, treatment and prevention), brucellosis practices (frequency of diagnosis, presence of reagents, types of samples for diagnosis, reporting practices and duration to receive feedback) and the FHWs attitude regarding brucellosis prevention and control (Appendix 1). In addition, knowledge on other zoonotic diseases and challenges during diagnosis and reporting of brucellosis were also explored. The questionnaire was prepared in English and translated to Swahili before been administered by members of the research team.

### Ethical consideration

Ethical clearance was granted by the Medical Research Coordinating Committee of the National Institute of Medical Research in the United Republic of Tanzania (NIMR/HQ/R.8a/vol. IX/3235). Permission to conduct the study in the selected districts was obtained from the National, regional and local government authorities and Local Government in Morogoro (AB.175/245/01/219) and Pwani (DCD.128/40/01/109) regions. Written informed consent was obtained from each study participant. Confidentiality and identity protection were ensured throughout the analysis and interpretation of the study findings.

### Data analysis

The collected data was submitted on daily basis to the protected server located at the Sokoine University of Agriculture, Morogoro, Tanzania. Data were exported from *AfyaData* app to the Microsoft excel sheet where they were cleaned then coded then exported to into Statistical Package for Social Sciences (SPSS) version 20 software for analysis. Descriptive analysis for frequencies, percentages and proportions was carried out. Knowledge and attitude of the participants were assessed using 5-point Likert scale, (strongly agree, agree, neutral, disagree and strongly disagree) that were measured using scoring method ranging from 5 to 1. The scores of the items were summed up and the total divided by number of the items giving a mean score for the each KAP domain as used in a previous study [31]. Knowledge was assessed using 36 Likert questions. The overall score for Likert questions in knowledge was 180 i.e. (36*5). The composite score was dichotomized using the mean obtained from the data (i.e., 90). Attitude was assessed using nine Likert questions giving the overall score of 45 i.e. (9*5), the mean obtained was 22.5. In assessment of practice related to brucellosis diagnosis and control, ten questions were measured by scoring method as follows: *yes,* option one score and *no* option zero score, *frequently* option two scores, *rarely* option one score and *none at all* option zero score. The total score for practice was 16. The mean score obtained was eight. Participants who scored above and or equal to the mean were regarded as knowledgeable and those scoring less than the mean were regarded as having poor knowledge. Awareness on other zoonotic diseases and challenges in reporting and diagnosis were assessed by binary variables which were Yes/No. Categorical variables were described by number and percent (N, %), while continuous variables were described by mean and standard deviation (Mean, SD). Chi-square and/fisher exact test were used to compare between categorical variables. A *p*-value of less than or equal to 0.05 was considered significant.

## Results

### Socio demographic characteristics of the participants

A total of 64 frontline health workers were enrolled in the study. Majority of participants 41(64%) were men whilst 23 (35.9%) were women. More than one third of the study population (40.6%) had college education. The participants’ age ranged from 21 to 67 with an overall mean of 39.7 (±1.38) years. The length of stay in a position ranged from 0 to 40 years with the majority having more than 5 years’ experience in work station. The socio-demographic characteristics of study participants are summarized in Table 1.

**Table 1:** Participant’s socio demographic characteristics in Kilosa and Chalinze districts

Variable		Chalinze (***N*** = 32)		Kilosa (***N*** = 32)	
	Total (***N*** = 64)	Frequency	Percentage	Frequency	Percentage
**Sex**					
Female	23	12	37.5	11	34.4
Male	41	20	62.5	21	65.6
**Age in years**					
21–30	19	6	18.8	13	40.6
31–40	18	13	40.6	5	15.6
41–50	15	7	21.9	8	25
> 50	12	6	18.8	6	18.8
**Education level**					
No education	3	2	6.3	1	3.1
Incomplete primary	1	1	3.1	0	0
Primary	23	12	37.5	11	34.4
Secondary	11	2	6.3	9	28.1
College	26	15	46.9	11	34.4
**Position**					
Medical officer	6	1	3.1	5	15.6
Medical Assistant	18	10	31.3	8	25
Nurse	6	3	9.4	3	9.4
Midwife	2	2	6.3	0	0
Community health worker	32	16	50	16	50
**Length of stay in position (years)**					
0–10	34	20	62.5	14	43.8
11–20	13	7	21.9	6	18.8
21–30	11	3	9.4	8	25
31–40	6	2	6.3	4	12.5
**Working experience in workstation**					
Less than a year	6	3	9.4	4	12.5
1–5 years	26	19	59.4	20	62.5
> 5 years	32	10	31.3	8	25.0

### HWs knowledge regarding brucellosis in Kilosa and Chalinze districts

In both Kilosa and Chalinze districts, 15 (93.8%) HWs in each district reported that they were aware of brucellosis, with four (25.0%) from Kilosa and seven (21.9%) from Chalinze, knowing the causative agent. Training programs regarding zoonotic diseases were reported as the main source of brucellosis knowledge among the participants in both Kilosa (68.8%) and Chalinze (87.5%) districts. A total of 14 (87.5%) HWs from Kilosa and ten (62.5%) in Chalinze strongly agreed that brucellosis can be transmitted from cattle to human while only four of them (25.0%) from Chalinze and none from Kilosa agreed to that statement. A total of 13 (81.3%) HWs in Kilosa and ten (62.5%) in Chalinze strongly agreed that drinking unpasteuralized milk can transmit brucellosis to humans while three (18.8%) in Chalinze and one (6.2%) HW in Kilosa agreed to this statement. Although majority of HWs heard about brucellosis only 15 (46.9%) were knowledgeable regarding brucellosis transmission, symptoms, diagnosis and prevention in both districts. Also, the level of awareness was significantly higher to the HWs within the age group of 21–30 years (*p* = 0.016) compared to other age groups. However, the level of knowledge didn’t differ with HWs gender, education and experience in position and workstation (*p* > 0.05) (Table 2).

**Table 2:** Association between sociodemographic factors and overall knowledge, attitude and practice scores of the healthcare workers (*N* = 32)

Variable	Knowledge (%)			Practice (%)			Attitude (%)		
	Poor	Good	***P***-value	Poor	Good	***P***-value	Negative	Positive	***P***-value
**Sex**									
Female	6(35.3)	2(13.3)	0.229	3(17.6)	5(33.3)	0.423	3(33.3)	5(21.7)	0.654
Male	11(64.7)	13(86.7)		14(82.4)	10(66.7)		6(66.7)	18(78.3)	
**Age in years**									
21–30	8(47.1)	6(40.0)	0.016*	8(47.1)	6(40.0)	0.954	2(22.2)	12(52.2)	0.428
31–40	8(47.1)	2(13.3)		5(29.4)	5(33.3)		4(44.4)	6(26.1)	
41–50	0(0.0)	5(33.3)		2(11.8)	3(20.0)		2(22.2)	3(13.0)	
> 50	1(5.9)	2(13.3)		2(11.8)	1(6.7)		1(11.1)	2(8.7)	
**Education level**									
Secondary	4(23.5)	2(13.3)	0.659	4(23.5)	2(13.3)	0.659	1(11.1)	5(21.7)	0.648
College	13(76.5)	13(86.7)		13(76.5)	13(86.7)		8(88.9)	18(78.3)	
**Position**									
Medical officer	9(52.9)	9(60.0)	0.467	8(47.1)	10(66.7)	0.179	4(44.4)	14(60.9)	0.164
Medical Assistant	2(11.8)	4(26.7)		5(29.4)	1(6.7)		2(22.2)	4(17.4)	
Nurse	4(23.5)	2(13.3)		4(23.5)	2(13.3)		1(11.1)	5(21.7)	
Midwives	2(11.8)	0(0.0)		0(0.0)	2(13.3)		2(22.2)	0(0.0)	
**Length of stay in position (years)**									
0–10	14(82.4)	9(60.0)	0.14	11(64.7)	12(80.0)	0.316	5(55.6)	18(78.3)	0.445
11–20	3(17.6)	3(20.0)		5(29.4)	1(6.7)		3(33.3)	3(13.0)	
21–30	0(0.0)	3(20.0)		1(5.9)	2(13.3)		1(11.1)	2(8.7)	
**Working experience in workstation (years)**									
< 1 year	3(17.6)	1(6.7)	0.795	3(17.6)	1(6.7)	0.795	0(0.0)	4(17.4)	
1–5 years	13(76.5)	13(86.7)		13(76.5)	13(86.7)		9(100)	17(73.9)	
> 5 years	1(5.9)	1(6.7)		1(5.9)	1(6.7)		0(0.0)	2(8.7)	

*Chi-square /Fisher Exact test significance at < 0.05

### HWs reporting and detection practices of brucellosis in Kilosa and Chalinze

Four (25.0%) HWs in Kilosa reported to advise patients regarding brucellosis testing, while none of the HWs in Chalinze reported providing advice to patients. On frequency of considering brucellosis during patients’ clinical assessment, one HW in Kilosa (6.3%) mentioned frequently and four (25.0%) HWs mentioned rarely in Kilosa, while only five HWs (31.2%) in Chalinze mentioned they rarely consider brucellosis for clinical assessment. A total of 11(68.8%) of HWs did not consider brucellosis at all in the clinical assessment in each of the districts. Overall, practice scores showed that 15 (46.9%) HWs had good practice regarding brucellosis prevention in both districts. Majority of males HWs were found to have good practices regarding brucellosis control than females. None of the sociodemographic factors were statistically significant with the level of practice (Table 2).

### HWs’ attitude on brucellosis prevention and control in Kilosa and Chalinze

A total of 15 (93.8%) HWs in Kilosa and 11 (68.8%) in Chalinze strongly agreed that inadequate knowledge and awareness among healthcare workers contributed to the misdiagnosis of brucellosis while only four (25.0%) in Chalinze and none in Kilosa agreed to this statement. Majority of HWs in Kilosa 14(87.5%) and 11(68.8%) in Chalinze strongly agreed that collaboration strategy in human and animal sector could prevent and control brucellosis, while only five (15.6%) in both districts agreed with this statement. Positive attitude regarding brucellosis prevention and control was recorded in 23 (71.9%) HWs in both districts, but it didn’t differ with age, gender, education or the length of stay in their workstation (*p* > 0.05). Majority of medical officers 14(60.9%) had positive attitude than other HWs and those with college education were found to had high positive attitude regarding brucellosis prevention than those with secondary education (Table 2).

### CHWs knowledge regarding brucellosis in Kilosa and Chalinze districts

Awareness of brucellosis was recorded in two (12.5%) CHWs in Kilosa and seven (43.8%) in Chalinze. Four CHWs in Kilosa (25.0%) and one (6.3%) in Chalinze reported friends as their main source of knowledge, while three (9.4%) in both districts reported radio, television, and newspapers as their sources. Only one CHW from Chalinze reported college and internet as the source of knowledge. One CHW from Kilosa (6.3%) and four (25.0%) from Chalinze strongly agreed that eating uncooked meat can cause brucellosis in humans while only one (6.3%) in Chalinze district agreed to that statement. Three (9.4%) of the CHWs in both districts strongly agreed that brucellosis could be transmitted from cattle to humans, while only two (6.2%) in both districts agreed with this statement. The overall knowledge scores showed that nine (28.1%) CHWs were knowledgeable regarding brucellosis transmission, symptoms, prevention and control measures in both districts. However, no significant difference was observed in their sociodemographic characteristics (Table 3).

**Table 3:** Association between sociodemographic factors and the mean score in knowledge, practice and attitude of the community health workers (*N* = 32)

Variable	Knowledge (%)			Practice (%)			Attitude (%)		
	Poor	Good	***P***-value	Poor	Good	***P***-value	Negative	Positive	***P***-value
**Sex**									
Female	12(52.2)	3(33.3)	0.444	14(50.0)	1(25.0)	0.603	12(48.0)	3(42.9)	1.000
Male	11(47.8)	6(66.7)		14(50.0)	3(75.0)		13(52.0)	4(57.1)	
**Age in years**									
21–30	2(8.7)	3(33.3)	0.309	5(17.9)	0(0.0)	0.251	2(8.0)	3(42.9)	0.028*
31–40	7(30.4)	1(11.1)		7(25.0)	1(25.0)		7(28.0)	1(14.3)	
41–50	8(34.8)	2(22.2)		7(25.0)	3(75.0)		10(40.0)	0(0.0)	
> 50	6(26.1)	3(33.3)		9(32.1)	0(0.0)		6(24.0)	3(42.9)	
**Education level**									
No formal education	3(13.0)	0(0.0)	0.085	2(7.1)	1(25.0)	0.508	3(12.0)	0(0.0)	0.024*
Incomplete primary	0(0.0)	1(11.1)		1(3.6)	0(0.0)		0(0.0)	1(14.3)	
Primary	18(78.3)	5(55.6)		20(71.4)	3(75.0)		20(80.0)	3(42.9)	
Secondary	2(8.7)	3(33.3)		5(17.9)	0(0.0)		2(8.0)	3(42.9)	
**Experience in position (years)**									
0–10	7(30.4)	4(44.4)	0.309	10(35.7)	1(25.0)	0.324	8(32.0)	3(42.9)	0.39
11–20	7(30.4)	0(0.0)		6(21.4)	1(25.0)		7(28.0)	0(0.0)	
21–30	5(21.7)	3(33.3)		8(28.6)	0(0.0)		5(20.0)	3(42.9)	
31–40	4(17.4)	2(22.2)		4(14.3)	2(50.0)		5(20.0)	1(14.3)	
**Working experience in workstation**									
< 1 year	3(13.0)	0(0.0)	0.616	3(10.7)	0(0.0)	0.74	3(12.0)	0(0.0)	0.576
1–5 years	8(34.8)	5(55.6)		12(42.9)	1(25.0)		9(36.0)	4(57.1)	
> 5 years	12(52.2)	4(44.4)		13(46.4)	3(75.0)		13(52.0)	3(42.9)	

*Chi-square test/fisher exact significance at < 0.05

### CHWs reporting practices and detection of brucellosis in Kilosa and Chalinze districts

A total of four (25.0%) CHWs in Chalinze reported advising farmers to consult veterinarians for brucellosis testing in animals, while none reported this practice in Kilosa. Only one (6.3%) of CHW in Chalinze mentioned recording brucellosis cases of animals and humans. Phones and notebooks were the main recording materials mentioned. Only three (18.8%) CHWs in Chalinze were found to report brucellosis cases using digital technology to submit reports to the higher levels, while in Kilosa reports were neither reported nor submitted. The overall practice scores showed that four out of 32 CHWs (12.5%) were found to have good practice regarding brucellosis prevention in both districts. However, no difference in practice level was observed in any of the CHWs sociodemographic characteristics (Table 3).

### CHWs attitude on brucellosis prevention and control in Kilosa and Chalinze

Seven of the CHWs (43.6%) in Chalinze and two (12.5%) in Kilosa strongly agreed that the use of guidelines/reference materials enhanced proper diagnosis and management of brucellosis. A total of six (37.5%) CHWs in Chalinze and two (12.5%) in Kilosa strongly agreed that engaging CHWs enhanced quick detection of brucellosis in humans, while none of the CHWs in both districts agreed with this statement. A total of six (37.5%) CHWs in Chalinze and two (12.5%) in Kilosa strongly agreed that public awareness is important for preventing the disease, whilst only one CHW (6.2%) in Chalinze and none in Kilosa agreed with the statement. Overall, seven CHWs had positive attitude towards brucellosis prevention. Community health workers within the age group of 21–30 and > 50 years were more likely to have a positive attitude towards brucellosis prevention than other age groups (*p* = 0.028). Community health workers with primary and secondary education (*p* = 0.024) were found to have a positive attitude regarding brucellosis prevention compared to those with no formal education (Table 3).

### HWs and CHWs’ awareness on other common zoonotic diseases in Kilosa and Chalinze districts

**Fig. 2:**
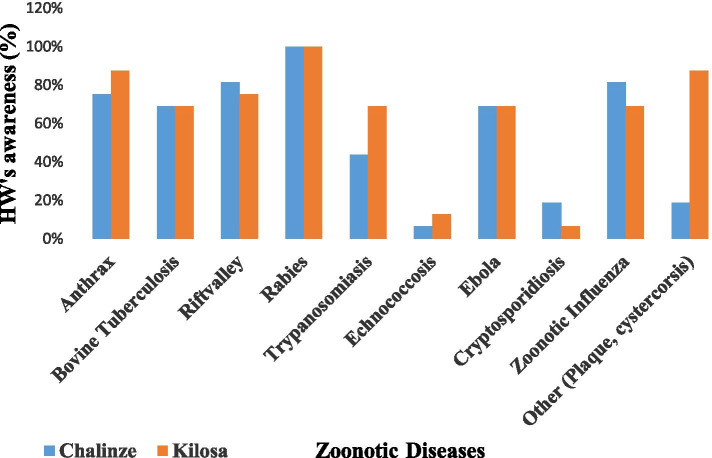
Awareness of healthcare workers (HWs) on other zoonotic diseases in Kilosa and Chalinze districts

**Fig. 3:**
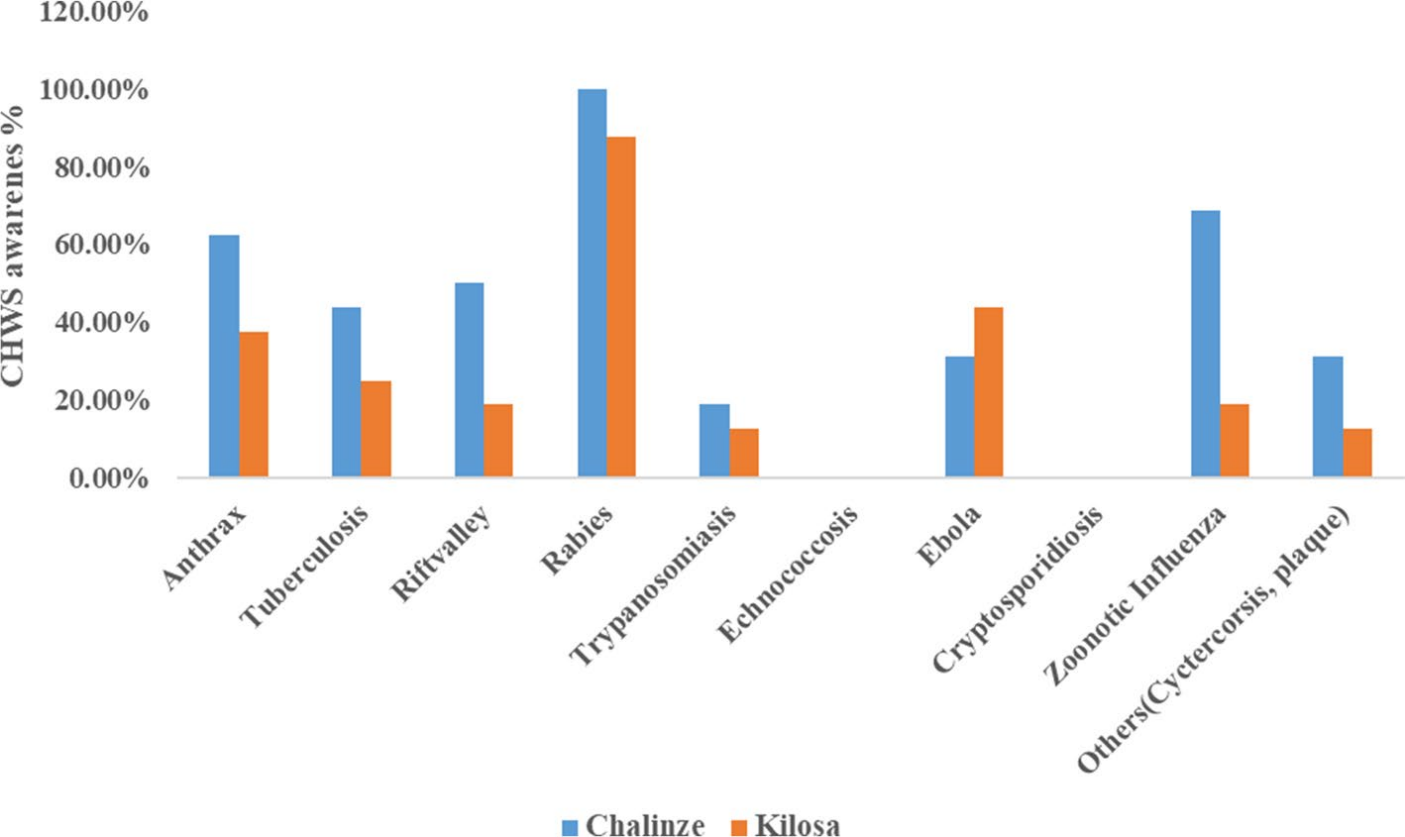
Community health workers’(CHWs) awareness on other zoonotic diseases in Kilosa and Chalinze districts

All (100%) HWs in both districts were found to have heard about rabies. Only two (12.5%) HWs in Kilosa and one (6.3%) in Chalinze reported to be aware of echinococcosis (Fig. 2). All CHWs (100%) in Chalinze and 14 (87.5%) in Kilosa, reported to have heard about rabies. None of the CHWs in both districts reported to be aware of echinococcosis and cryptosporidiosis (Fig. 3).

### Challenges on diagnosing and reporting brucellosis in Kilosa and Chalinze districts

In both districts, 29 (90.6%) of HWs reported inadequate knowledge and skills on brucellosis diagnosis as the main challenge they faced. Nine (28.1%) CHWs in both districts mentioned lack of knowledge and skills as the main challenge they faced in the detection of clinical manifestations related to brucellosis (Table 4).

**Table 4:** Challenges regarding detection and reporting of brucellosis in Kilosa and Chalinze districts

	Kilosa, n (16)	Chalinze, n (16)
	n (%)	n (%)
**Healthcare workers**		
Inadequate knowledge and skills	14(87.5)	15(93.8)
Lack of diagnostic resources	14(87.5)	15(93.8)
Inadequate human resources	5(31.3)	13(81.3)
Unspecific nature of clinical signs	9(56.3)	15(93.8)
Inadequate community awareness	10(62.5)	15(93.8)
Late presentation of cases to health facility	8(50.0)	11(68.8)
Inadequate collaboration with relevant sectors	8(50.0)	12(75.0)
Drug deficiency in the health facilities	4(25.0)	7(43.6)
Ineffective drug to treat the diseases	1(6.3)	7(43.6)
**Community health workers**		
Inadequate knowledge and skills	2(12.5)	7(43.6)
Long distance to the health facilities	1(6.3)	0(0.0)
Unreliable transport	2(12.5)	0(0.0)
Inadequate community awareness	2(12.5)	2(12.5)
Late presentation of cases to health facility	0(0.0)	1(6.3.0)

## Discussion

This study assessed the knowledge, attitude and practices (KAP) of healthcare workers (HWs) and community health workers (CHWs) regarding brucellosis in the selected pastoral districts. The study findings showed clearly that the majority of participants had limited knowledge on human brucellosis. The present study revealed a reasonable level of knowledge and awareness regarding brucellosis transmission, symptoms, diagnosis, and preventive measures among HWs in both districts. Although the majority (93.8%) of HWs were aware of the disease, very few (34.4%) knew its causative agent. Similar findings were found in a study conducted in Uganda which reported health workers lack of awareness of the causative organism and transmission route of brucellosis leading to poor diagnosis and improper treatment [26]. These findings are similar to those reported by Mbaipago (2020) in Chad, who found that 72% of HWs were aware of brucellosis [32]. This suggests that HWs awareness on zoonotic diseases varies, and there is a need to provide health education regarding zoonoses, especially brucellosis. The current study also, revealed a statistically significant difference between overall knowledge and HWs age. HWs within the 21–30 years age group were found to be more knowledgeable (*p* = 0.016) regarding brucellosis than other age groups. This may be due to the fact that majority were newly employed, with better recall of their professional training. Contrary results were found in a study conducted in Sudan, which found participants within the 21–30 years age group had poor knowledge regarding brucellosis [31].

More than three quarters of the HWs had college education while the majority of the CHWs had primary education. The low education level of CHWs might have an effect on the awareness and practices towards management of brucellosis in pastoral communities. Similar results were found in a KAP studies conducted in Sudan and Northern Uganda for animal health workers, medical and community workers in 2017 which reported nearly half of the study participants were of the age group between 21 and 30 years [26, 31].

The present study revealed a low awareness among the CHWs regarding brucellosis in both districts. It was found that only (28.1%) CHWs heard about brucellosis in the two districts. The poor knowledge among CHWs in both districts, could be attributed to inadequate public health promotion (especially regarding zoonotic diseases). This observation may contribute to poor/late detection of clinical manifestations suggestive of brucellosis at the community level. These findings corroborate the study conducted in China [33] and South Africa [34] that reported poor understanding of brucellosis and risky practices among the CHWs but are contrary with previous studies conducted in Kenya [35], Uganda [11] and Tajikistani [22] which reported higher proportions of CHWs awareness of brucellosis. This study indicated that workshops and refresher courses are important in raising brucellosis awareness among CHWs.

The main sources of brucellosis knowledge reported among the HWs in this study were training programs. Other sources mentioned included newspapers, television, radio, veterinarians and health workers. The main source of knowledge of brucellosis for CHWs was the health workers working in the respective village dispensaries and health centers. Also, media and friends were another source of information. As a result, the knowledge of brucellosis did not differ significantly at the community level. These findings were similar to those reported in other studies conducted in different places such as in South Africa [34], Kenya which found main knowledge sources were veterinarians and health workers [35], radio, television and newspapers in West and Central Africa [36], and friends or coworkers in Tajikistan and Pakistan [22, 23]. The increasing number of local radios and television stations reaching the remote areas could be an opportunity to reach the CHWs and the general community with information regarding zoonoses as pointed out by some of the participants in this study.

The current study showed that HWs in both districts had an average awareness of diagnosis, prevention, and control of brucellosis. Nearly half (43.8%) of the HWs identified a history of fever as a clinical indication of infection, while other HWs agreed that patient’s history of exposure and serology results can be used for brucellosis diagnosis. This implies that HWs were aware of the techniques used to diagnosis brucellosis, but due to the absence of appropriate tools and reagents, they were unable to perform the diagnosis. Some of the common prevention measures of brucellosis mentioned by study participants included: boiling of milk, consumption of well-cooked meat and proper disposal of aborted materials. Similar results were found in the study conducted in Kenya [37], which reported boiling of milk as one of preventative measures, as well as proper cooking of meat/spleen and proper disposal of aborted materials.

The present study also revealed poor knowledge among CHWs in both districts of the prevention and control of brucellosis. The majority of CHWs (84.4%) were not aware that boiling milk can prevent *Brucella* infection. This may be due to the fact that the majority of the CHWs were not aware of the transmission routes of the disease. Similar findings were reported by a study conducted in the Central and Eastern zones of Tanzania on milk quality and health risks associated with consumption of milk from pastoral herds, which showed that the majority of herders were not aware of the health risks associated with milk consumption [38]. Also, the study showed that majority (81.3%) of CHWs were not aware that wearing gloves when handling animals and proper disposal of aborted materials can prevent brucellosis transmission. This indicated that the risk of infection of the community served by these CHWs is likely to increase due to lack of awareness of the disease. A similar observation was made in a KAP study conducted in India which found that most study participants did not use proper protective gear while handling animals or aborted materials [39].

The majority of HWs, mainly the medical officers and nurses had a general positive attitude regarding brucellosis control compared to midwives and assistant medical officers. Also, HWs with college education had a more positive attitude regarding brucellosis prevention than those with secondary education but the difference was not significant. Studies conducted in Northern Uganda and Sri Lanka for medical and community workers similarly reported HWs to have the positive attitude towards brucellosis control [26, 40]. The current study revealed that male CHWs were more positive in attitude regarding brucellosis prevention when compared to their female counterparts. Also, CHWs within the 21–30 and > 50 years age group were more likely to have positive attitude than the other age groups (*p* = 0.028). This observation may be due to the accumulation of experience and insight about disease in general that comes with age. The study also found that CHWs with secondary and primary education were more likely to have positive attitude compared to those with no formal education (*p* = 0.024), this indicates the higher risk of the disease to the community served by the less formally trained CHWs. Other studies in Kenya and South Western Uganda similarly reported community workers to have a negative attitude towards brucellosis control [11].

However, this good attitude didn’t translate into good practices across all the study groups. The bad practices recorded in this study were a great risk for human infection [26]. The study revealed that the reporting practices of brucellosis and frequency of considering brucellosis in diagnosis were very poor, in both districts. The frontline health workers did not report brucellosis cases often and this may be attributed to the fact that they did not consider it during diagnosis. Also, they were not well informed about the disease. The study found that health facilities didn’t have diagnostic kits for brucellosis. The absence of test kits at health facilities may indicate that brucellosis is still a neglected disease in Tanzania [24]. HWs tended to concentrate on other endemic diseases in their areas and ignored zoonoses as causes of illness probably due to narrow training curricula in medical training institutions that generally do no emphasize on zoonoses and brucellosis in particular [21]. Since the disease is malaria-like, the commonly prioritized diseases were malaria and, in some cases, tuberculosis and typhoid fever. These findings are similar to the studies among health workers conducted in northern Tanzania, Egypt and Uganda among medical, veterinary and community health workers that showed positive attitudes towards brucellosis which however, did not reflect in good practices of prevention and control [26, 41, 42]. The study also revealed that participants with higher formal education had good practices regarding brucellosis. These findings are similar to the study conducted in China (Arif, 2017) which reported participants with higher formal education level showed good practices in brucellosis prevention [33].

Furthermore, this study found that the frontline health workers’ knowledge on other zoonotic diseases was poor. Most of the participants in Kilosa and Chalinze reported to be more aware of rabies and bovine tuberculosis, but not echinococcosis, cryptosporidiosis or Ebola. This may be due to frequent mass campaigns of rabies conducted in most areas to control the disease such as mass dog vaccination campaigns. A similar observation was made in the KAP study of medical practitioners which found poor knowledge on echinococcosis but good knowledge on rabies done in northern Tanzania by Kunda et al [21] and other zoonoses. A similar KAP study on the community done in Tanzania by Sambo [43] found 96% of the community were aware of rabies. Lack of health personnel knowledge of disease manifestation and diagnosis may lead to frequent misdiagnosis.

The major challenges reportedly faced by participants during the diagnosis of brucellosis were limited knowledge and skills among the frontline health workers. The majority of HWs reported lack of diagnostic tools and reagents for brucellosis diagnosis as another challenge faced in both districts. This indicates the high risk of mis- or under-diagnosis of brucellosis in these communities. These findings were generally similar to those of a study conducted in South Western Uganda [11].

## Conclusion and recommendation

This study assessed knowledge, attitude and practices among frontline health workers. The majority of participants had poor knowledge of brucellosis and appropriate preventive measures, although they showed a positive attitude regarding brucellosis prevention. These findings highlighted the need for intensification of health education on brucellosis interventions strategies and the use of modern reporting technologies. Also, these results could provide guidance on formulation of strategies to improve early detection and management of brucellosis in the study districts and other similar settings. In Tanzania mobile technology has been implemented for reporting of other zoonoses to improve disease detection and surveillance among frontline health workers [44–46]. It would be of advantage if concerted efforts could be made to improve community knowledge on brucellosis as a zoonotic disease and to ensure that frontline healthcare practitioners are equipped to identify and treat the disease especially among older age groups and those with no formal education. A noted limitation of this study was in the limited and purposively selection of study sites based on brucellosis risk, suggesting that an attempt to generalize the findings should be made with caution.

## Supplementary Information

**Additional file 1 **: **Appendix 1**.Questionnaire used for the study. **Appendix 2**. Ethical consideration.

## Data Availability

The datasets used and/or analyzed during the current study are available from the corresponding author on reasonable request.

## Abbreviations

SPSS: Statistical package for the social sciences.
HWs: Healthcare workers
FHW: Frontline health workers
CHW: Community health workers
KAP: Knowledge, Attitude and Practices
SD: Standard deviation
