# Supplementary material for: Knowledge, attitude and practices of frontline health workers in relation to detection of brucellosis in rural settings of Tanzania: a cross-sectional study
Source: One Health Outlook. 2022 Jan 4;4:1. doi: 10.1186/s42522-021-00056-5 (PMC8725462; doi:10.1186/s42522-021-00056-5)

**APPENDICES**

**Appendix 1: Questionnaire**

District name…………………. Date of interview ……………………

1. **FACILITY DETAILS**
   1. Name of the facility………………….
   2. Type of health facility

- Dispensary
- Health center
- Hospital
  1. Ward name …………………….

1. **PARTICIPANTS DETAILS**
   1. Name of participant………………...
   2. Education

- Primary
- Secondary
- College/university
- No formal education
- Incomplete primary education
  1. Profession
- Medical assistant (clinical officer)
- Medical officer
- Assistant medical officer
- Medical doctor
- Other
  1. Position
- Officer in charge
- Clinician
- Other
  1. Number of months in your position…………………….
  2. Number of months in your work station……………………
  3. Sex
- Female
- Male

2.6. Age (years)…………………………

1. **GENERAL KNOWLEGDE ON BRUCELLOSIS**
   1. Have you heard of a disease called brucellosis?

- Yes
- No
  1. What organism causes brucellosis …………………...
  2. Where did you get informed about brucellosis?
- Training program
- Co-worker
- Veterinary officials
- Friends
- Radio/Television/newspaper
- Other

1. **Brucellosis transmission: On the scale shown, how much do you agree /disagree with the following statements**
   1. Brucellosis affects domestic ruminants (cattle, sheep, and goats)

- Strongly agree
- Agree
- Neither agree nor disagree (neutral)
- Disagree
- Strongly disagree
  1. brucellosis affects humans
- Strongly agree
- Agree
- Neither agree nor disagree (neutral)
- Disagree
- Strongly disagree
  1. brucellosis can be transmitted from cattle to humans
- Strongly agree
- Agree
- Neither agree nor disagree (neutral)
- Disagree
- Strongly disagree
  1. Brucellosis can be transmitted from sheep/goats to humans
- Strongly agree
- Agree
- Neither agree nor disagree (neutral)
- Disagree
- Strongly disagree
  1. A person could acquire brucellosis by drinking raw/unpasteurized milk
- Strongly agree
- Agree
- Neither agree nor disagree (neutral)
- Disagree
- Strongly disagree
  1. A person could acquire brucellosis by contact with aborted foetus or placenta
- Strongly agree
- Agree
- Neither agree nor disagree (neutral)
- Disagree
- Strongly disagree
  1. A person could acquire brucellosis by eating meat, liver or spleen not well cooked
- Strongly agree
- Agree
- Neither agree nor disagree (neutral)
- Disagree
- Strongly disagree
  1. Sexual transmission of brucellosis can occur in humans
- Strongly agree
- Agree
- Neither agree nor disagree (neutral)
- Disagree
- Strongly disagree
  1. A person can acquire brucellosis by inhaling aerosolized bacteria
- Strongly agree
- Agree
- Neither agree nor disagree (neutral)
- Disagree
- Strongly disagree
  1. A person can acquire brucellosis through breaks in the skin or mucous membrane
- Strongly agree
- Agree
- Neither agree nor disagree (neutral)
- Disagree
- Strongly disagree
  1. A person can acquire brucellosis through blood transfusion from infected person
- Strongly agree
- Agree
- Neither agree nor disagree (neutral)
- Disagree
- Strongly disagree
  1. A person can acquire brucellosis through contact with menstrual blood from the infected person
- Strongly agree
- Agree
- Neither agree nor disagree (neutral)
- Disagree
- Strongly disagree

1. **Clinical sign and symptoms of brucellosis in human:** **on the scale shown, how much do you agree /disagree with the following statements. The common clinical signs and symptoms of brucellosis include**
   1. Continuous or intermittent fever

- Strongly agree
- Agree
- Neither agree nor disagree (neutral)
- Disagree
- Strongly disagree
  1. fatigue
- Strongly agree
- Agree
- Neither agree nor disagree (neutral)
- Disagree
- Strongly disagree
  1. sweating
- Strongly agree
- Agree
- Neither agree nor disagree (neutral)
- Disagree
- Strongly disagree
  1. Loss of appetite
- Strongly agree
- Agree
- Neither agree nor disagree (neutral)
- Disagree
- Strongly disagree

5.5. muscular and joint pain

- Strongly agree
- Agree
- Neither agree nor disagree (neutral)
- Disagree
- Strongly disagree
  1. Pain in the back
- Strongly agree
- Agree
- Neither agree nor disagree (neutral)
- Disagree
- Strongly disagree
  1. Weight loss
- Strongly agree
- Agree
- Neither agree nor disagree (neutral)
- Disagree
- Strongly disagree
  1. Headache
- Strongly agree
- Agree
- Neither agree nor disagree (neutral)
- Disagree
- Strongly disagree
  1. coughing
- Strongly agree
- Agree
- Neither agree nor disagree (neutral)
- Disagree
- Strongly disagree
  1. Swollen lymph nodes
- Strongly agree
- Agree
- Neither agree nor disagree (neutral)
- Disagree
- Strongly disagree
  1. Abortion
- Strongly agree
- Agree
- Neither agree nor disagree (neutral)
- Disagree
- Strongly disagree
  1. Inflammation of the epididymis and/testicles
- Strongly agree
- Agree
- Neither agree nor disagree (neutral)
- Disagree
- Strongly disagree

1. Awareness on Diagnosis of brucellosis: on the scale shown, how much do you agree /disagree with the following statements. Diagnosis of brucellosis can be made through
   1. history of prolonged at least a week by presence of clinical signs like repeated fever

- Strongly agree
- Agree
- Neither agree nor disagree (neutral)
- Disagree
- Strongly disagree
  1. patient’s history of exposure to likely sources of the diseases
- Strongly agree
- Agree
- Neither agree nor disagree (neutral)
- Disagree
- Strongly disagree
  1. serological test
- Strongly agree
- Agree
- Neither agree nor disagree (neutral)
- Disagree
- Strongly disagree
  1. culture technique
- Strongly agree
- Agree
- Neither agree nor disagree (neutral)
- Disagree
- Strongly disagree
  1. **Diagnosis and reporting practices in healthcare workers**
  2. Do you advice clients/patients to get tested for brucellosis
- Yes
- No
  1. Do you provide any test that can be used to diagnose brucellosis in humans in this facility?

 Yes

 No

- 1. If yes to Qn 7.2 mention the test……………………
  2. What human samples do you take for diagnosis of brucellosis?
- Blood
- Cerebrospinal fluid
- Others
  1. How many cases of brucellosis have you ever suspected in patients who attended this facility during the past 1 month? …………………………..
  2. How many cases of brucellosis have you ever confirmed in patients who attended this facility during the past 1 month? …………………………….
  3. How frequently do you consider brucellosis diagnosis for patients attending this facility?
- Frequently
- Rarely
- None at all
  1. Do you have test reagents for brucellosis in this facility?
- Yes
- No
  1. If yes to Qn 7.8, what reagent do you have in place………………………
  2. Do you refer samples elsewhere for brucellosis testing?
- Yes
- No
  1. If you yes to Qn. 7.10, where do you refer …………….
  2. If you refer sample to testing elsewhere, do you get feedback on results
- Yes
- No
- NA
  1. How long it usually takes to receive resulting from referral point
- Within a day
- 2-3 days
- 4-7 days
- `1-2 weeks
- 3-4 weeks
- >month
- NA
  1. How do you receive results from referral point?
- Phone calls/sms
- Collected physically on paper
- Digital technology
- Not applicable
  1. Do you communicate results from referral point to patients?
- Yes
- No
  1. How do you communicate test result to patients? (1mark)
- Phone calls/sms
- Collected physically on paper
- Digital technology
- Not applicable
  1. Do you have guidelines for diagnosis and management of brucellosis?
- Yes
- No
  1. If yes to Qn 7.17, ask to see the guidelines: guidelines seen?
- Yes
- No

1. **Detection and Reporting practices in community health workers**

8.1. Do you advice /refer patients to visit health facilities when they experience clinical manifestation suggestive of brucellosis?

- Yes
- No
  1. How do you provide referral to health facility?
- Verbal
- Written
- Do not provide referral
  1. Do you advise farmers to consult veterinary officers when their animals experience clinical manifestation suggestive of brucellosis?
- Yes
- No
  1. Do you keep record of suspected brucellosis cases in animals?
- Yes
- No
  1. Do you keep record of suspected brucellosis cases in human?
- Yes
- No
  1. Where do you keep record?
- Note book
- Records forms
- Phone
- NA
  1. How frequently do you submit reports for human cases to higher levels?
- Daily
- Weekly
- Monthly
- Quarterly
- Other
- Not submitting
  1. How frequently do you submit reports for animal cases to higher levels?

 Daily

 Weekly

 Monthly

 Quarterly

 Other

 Not submitting

- 1. How do you submit reports to higher levels?
- Phone call/sms
- Physical visitation
- Digital technology
- Other
  1. How long it usually takes to receive feedback from higher level?
- Within a day
- 2-3 days
- 4-7 days
- 1-2 weeks
- 3-4 weeks
- >month
- Never receive feedback
  1. How do you receive feedback from higher level?

 Phone call/sms

 Physical visitation

 Digital technology

 Other

- 1. How do you communicate feedback to patients/community members?
- Phone call/sms
- Physical visitation
- Digital technology
- Gathering/meeting
- Not communicating
  1. Have ever receive training on the detection of diseases transmissible between animals and humans?
- Yes
- No
  1. Do you have guidelines or references materials for detection, recording and submission of clinical manifestation suggestive of brucellosis?
- Yes
- No
  1. If yes to last Qn; ask to see guidelines/references materials: seen
- Yes
- No

1. **Prevention and control of brucellosis**: **on the scale shown, how much do you agree /disagree with the following statements. Brucellosis can be prevented by:**

9.1. Drinking boiled/pastoralized milk

- Strongly agree
- Agree
- Neither agree nor disagree (neutral)
- Disagree
- Strongly disagree
  1. Eating meat, liver or spleen well cooked
- Strongly agree
- Agree
- Neither agree nor disagree (neutral)
- Disagree
- Strongly disagree
  1. Control of brucellosis in human depends much on control of the disease in animals
- Strongly agree
- Agree
- Neither agree nor disagree (neutral)
- Disagree
- Strongly disagree
  1. Wearing gloves when handling aborted fetuses or placenta
- Strongly agree
- Agree
- Neither agree nor disagree (neutral)
- Disagree
- Strongly disagree
  1. Washing hands after close contact with animals and/their products
- Strongly agree
- Agree
- Neither agree nor disagree (neutral)
- Disagree
- Strongly disagree
  1. Proper disposal of infected materials
- Strongly agree
- Agree
- Neither agree nor disagree (neutral)
- Disagree
- Strongly disagree

1. **Attitude: on the scale shown, how much do you agree /disagree with the following statements.**

10.1. Brucellosis is an important public health disease in Tanzania

- Strongly agree
- Agree
- Neither agree nor disagree (neutral)
- Disagree
- Strongly disagree
  1. Brucellosis affects mostly the pastoral communities
- Strongly agree
- Agree
- Neither agree nor disagree (neutral)
- Disagree
- Strongly disagree
  1. Brucellosis can be successfully cured
- Strongly agree
- Agree
- Neither agree nor disagree (neutral)
- Disagree
- Strongly disagree
  1. Inadequate knowledge and awareness of brucellosis amongst the FHWs contribute to misdiagnosis of the disease
- Strongly agree
- Agree
- Neither agree nor disagree (neutral)
- Disagree
- Strongly disagree
  1. Brucellosis can be prevented and controlled through collaborative strategy between animal and human sectors
- Strongly agree
- Agree
- Neither agree nor disagree (neutral)
- Disagree
- Strongly disagree
  1. Use of guidelines or references materials can be enhanced appropriate diagnosis and management of brucellosis
- Strongly agree
- Agree
- Neither agree nor disagree (neutral)
- Disagree
- Strongly disagree
  1. Engagement of community health workers in event-based surveillance can enhanced early detection and reporting of brucellosis suspected cases
- Strongly agree
- Agree
- Neither agree nor disagree (neutral)
- Disagree
- Strongly disagree
  1. Public awareness is important for disease prevention and control
- Strongly agree
- Agree
- Neither agree nor disagree (neutral)
- Disagree
- Strongly disagree
  1. Use of mobile phone-based technology can enhance early detection and communication of test results compared with paper-based system
- Strongly agree
- Agree
- Neither agree nor disagree (neutral)
- Disagree
- Strongly disagree

1. **What other disease transmissible between animals and humans: are u aware of?**

**11.1 Anthrax: Awareness**

- Yes
- No
  1. **Bovine tuberculosis: awareness**
- Yes
- No
  1. **Rift valley: awareness**

Yes

No

- 1. **Rabies: awareness**
- Yes
- No
  1. **Trypanosomiasis**
- Yes
- No
  1. **Echinococcosis: awareness**
- Yes
- No
  1. **Ebola: awareness**
- Yes
- No
  1. **Cryptosporidiosis: awareness**
- Yes
- No
  1. **Zoonotic influenza: awareness**
- Yes
- No

Other zoonotic diseases: ……………………

1. **Challenges: what are the challenges the FHWs face in the diagnosis and management of brucellosis**

12.1. Inadequate knowledge and skills Yes/No

12.2. Inadequate/ lack of diagnostic resources Yes/No

12.3. Inadequate human resources Yes/No

12.4. Unspecific nature of clinical signs and symptoms of the disease Yes/No

12.5. Inadequate awareness in the community Yes/No

12.6. Late presentation of the cases to health facility Yes/No

12.7. Inadequate collaboration with other relevant sectors Yes/No

12.8. Drug deficiency Yes/No

12.9. Ineffective drugs Yes/No

12.10. Long distance to the health facilities Yes/No

12.11. Unreliable transport Yes/No

12.12. Use of paper-based system Yes/No

12.13. Other

13. What type of mobile phone do you own

- Ordinary phone
- Smart phone
- None

Appendix 2: Ethical consideration


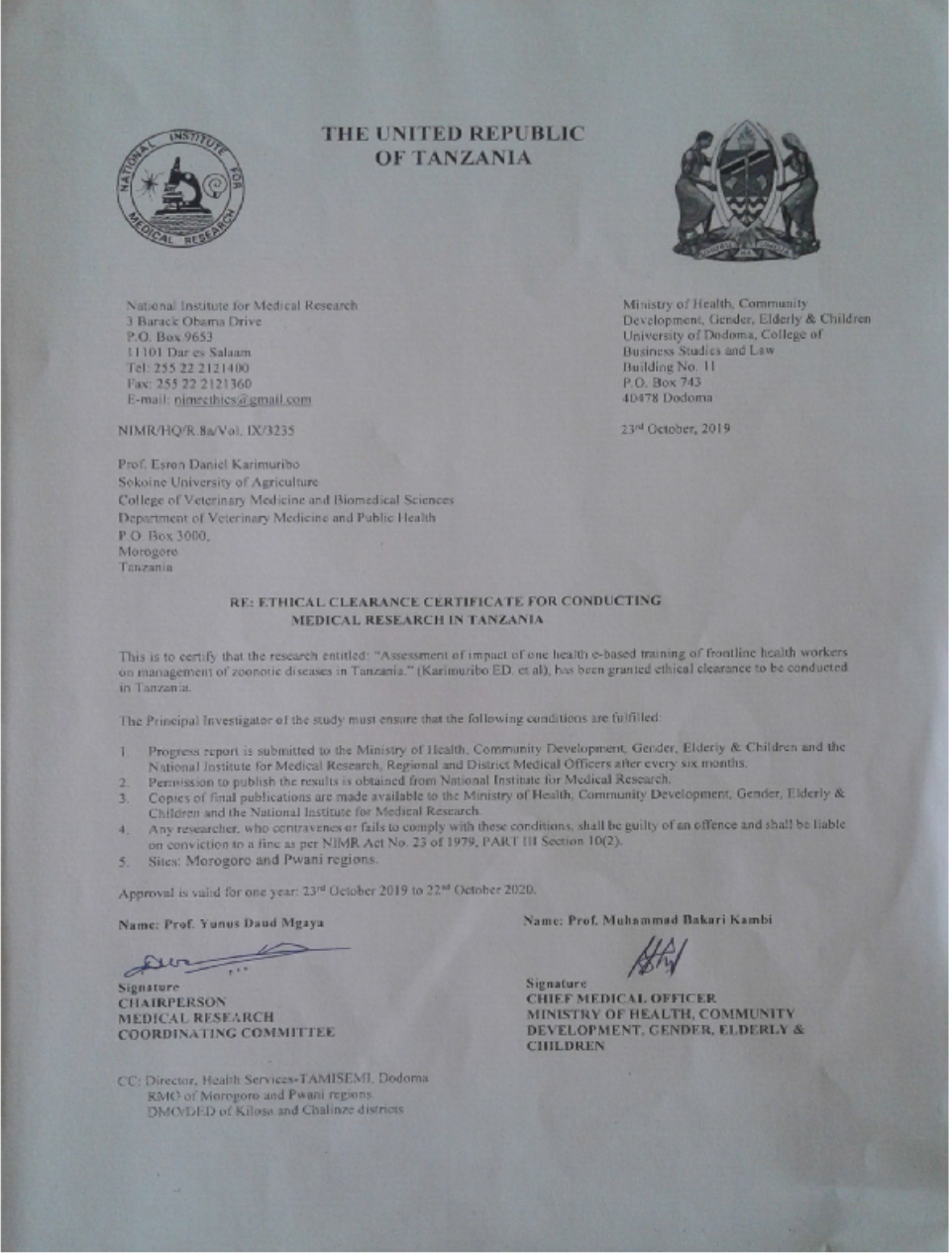

Supplement: Supplementary file 1 — Additional file 1 : Appendix 1.Questionnaire used for the study. Appendix 2. Ethical consideration. [file 42522_2021_56_MOESM1_ESM.docx]
